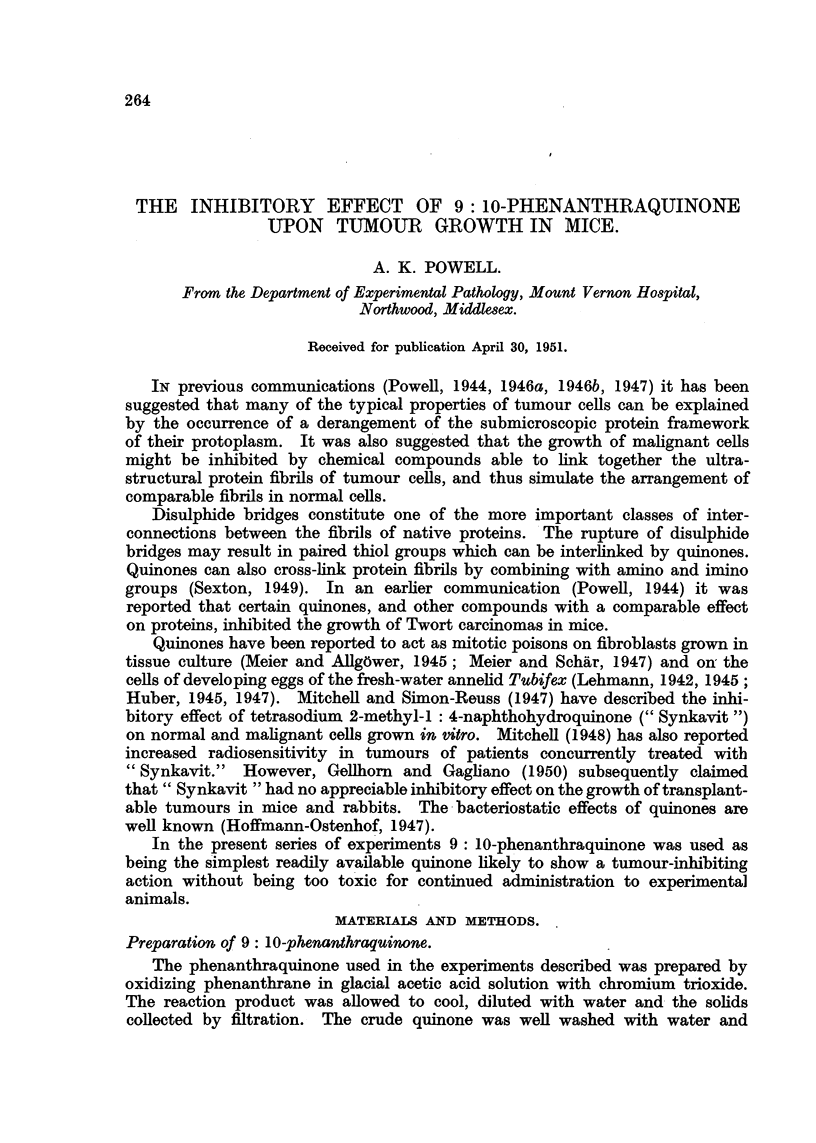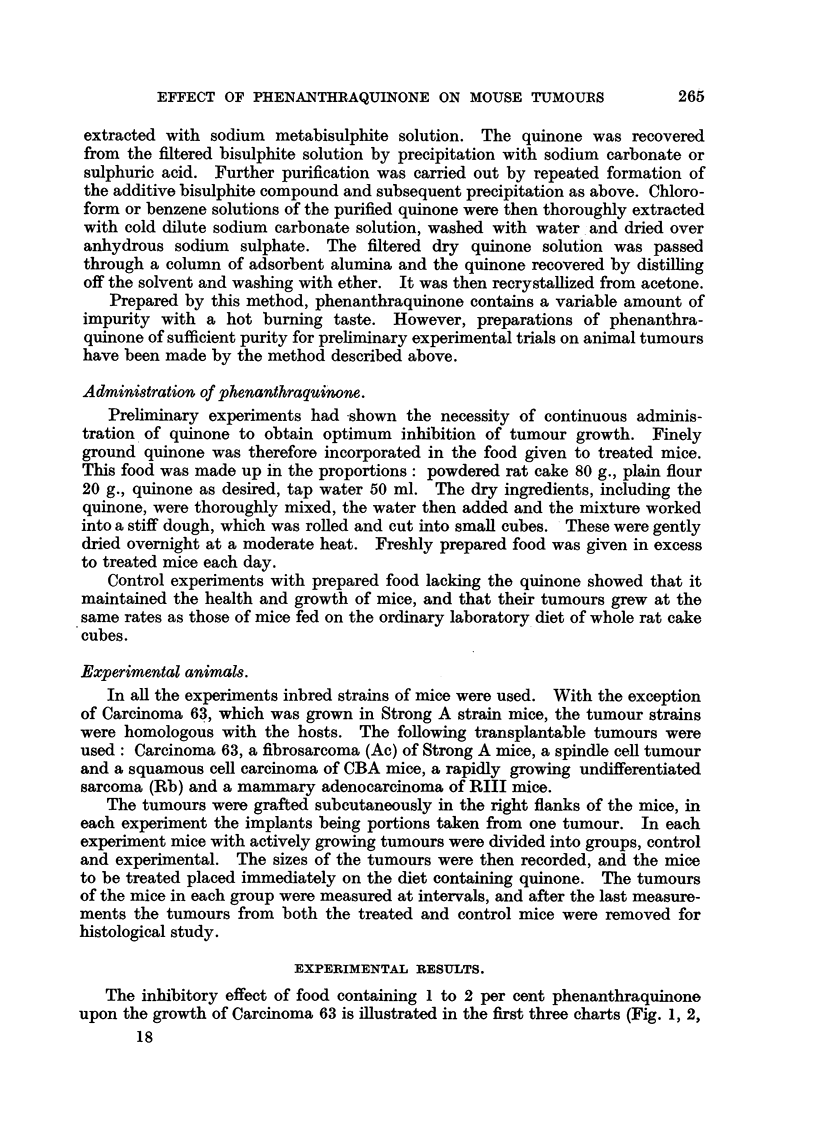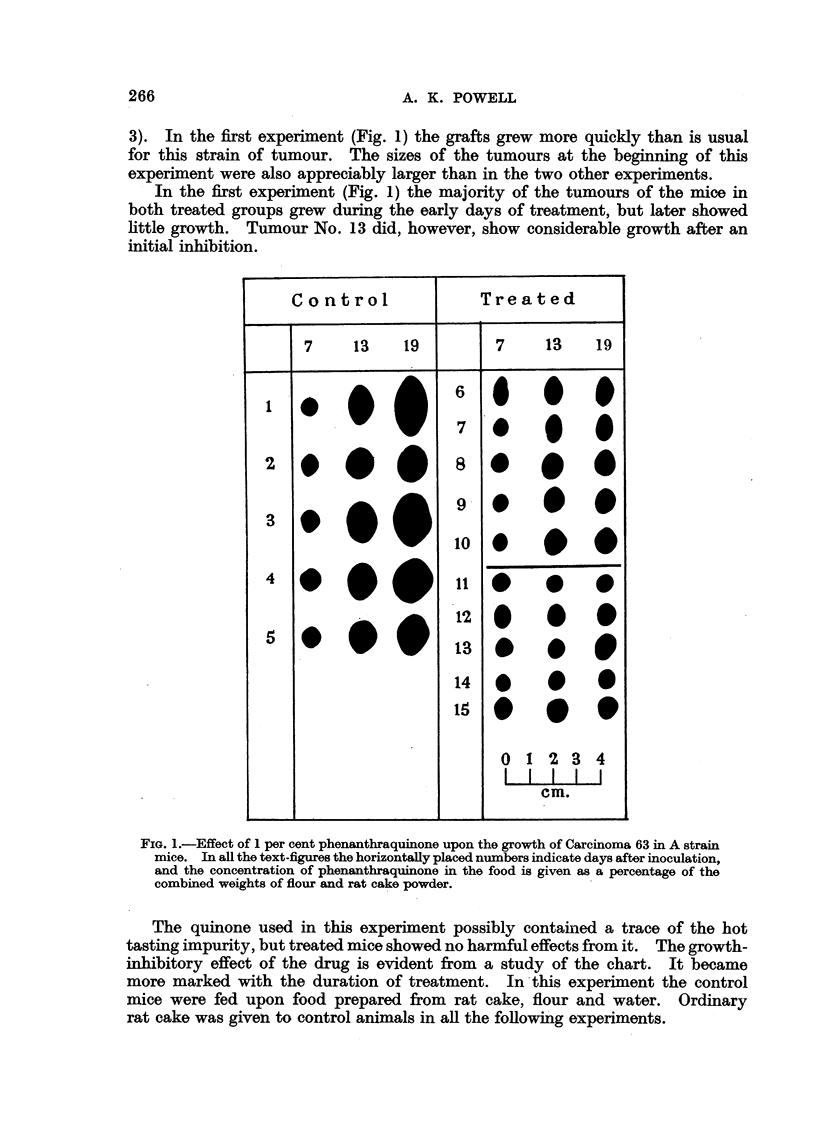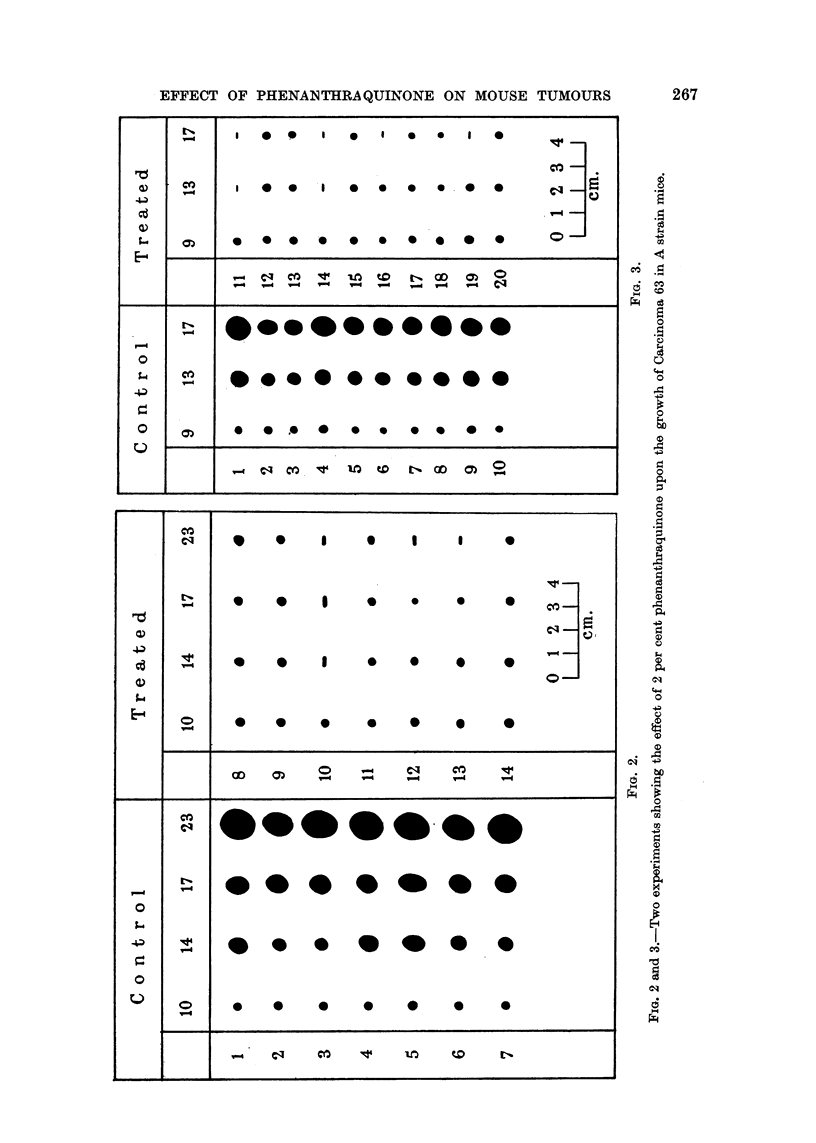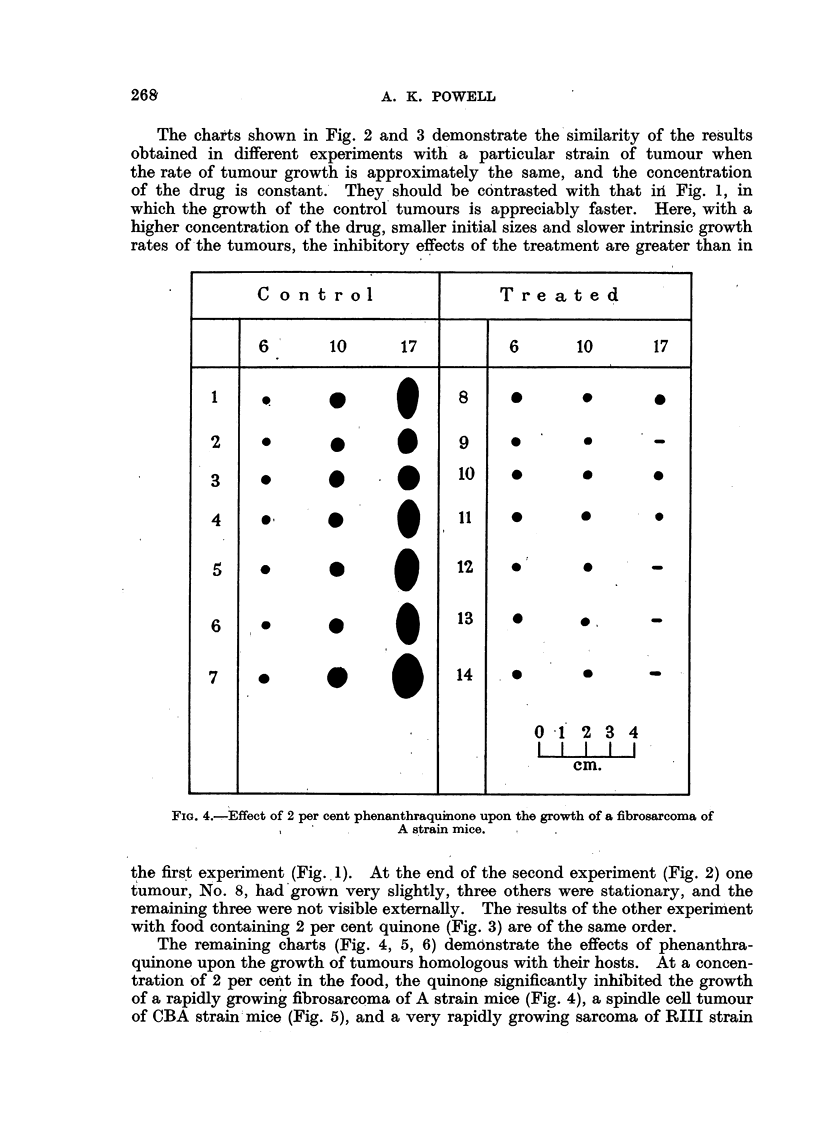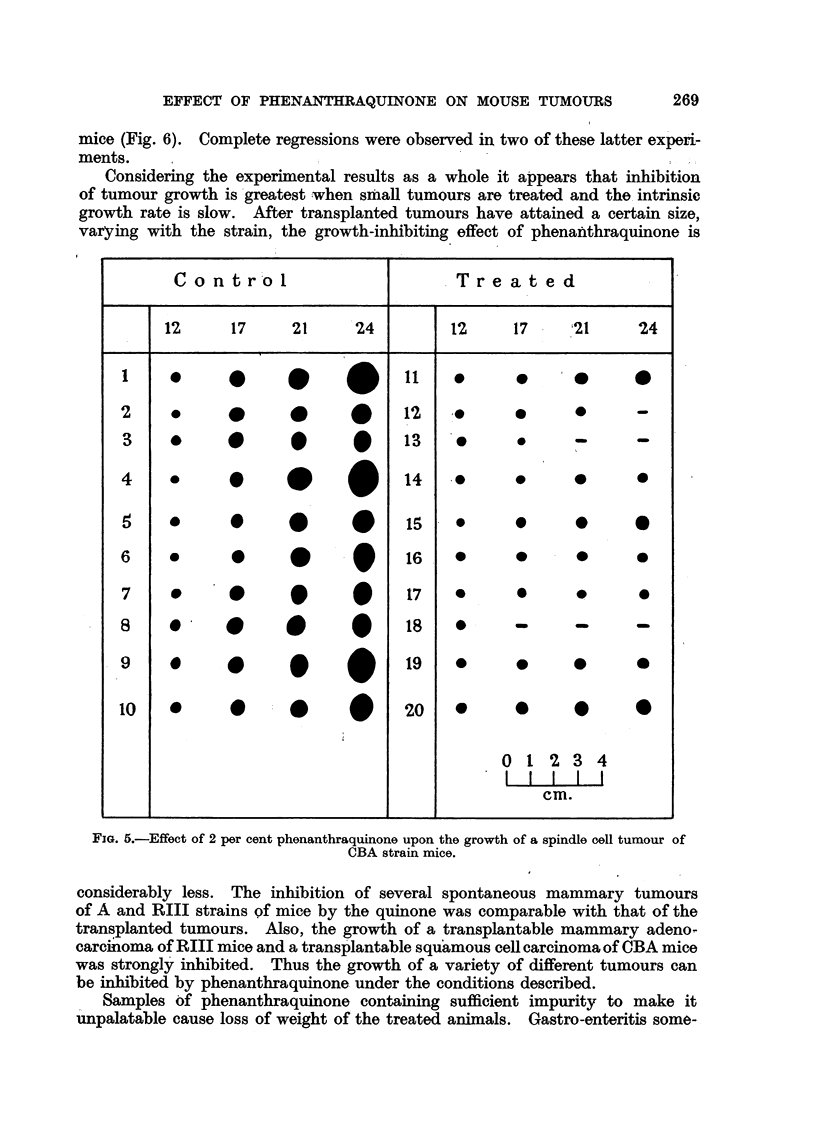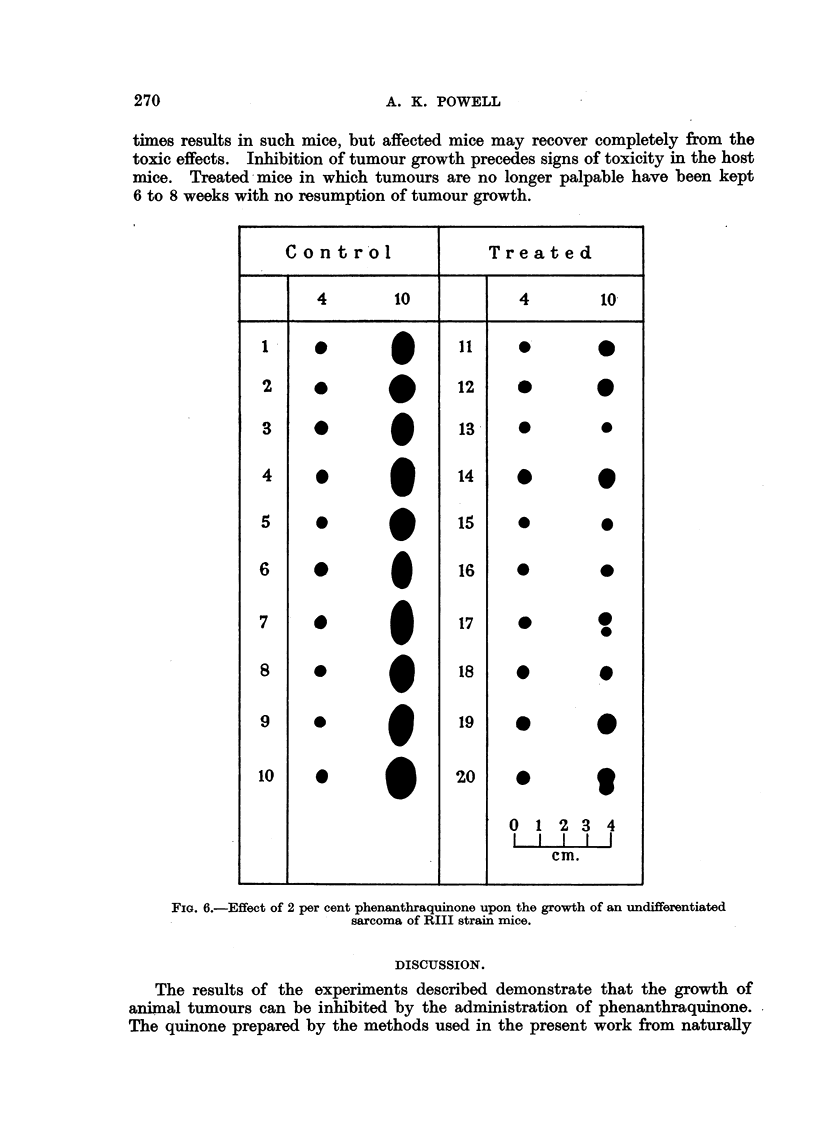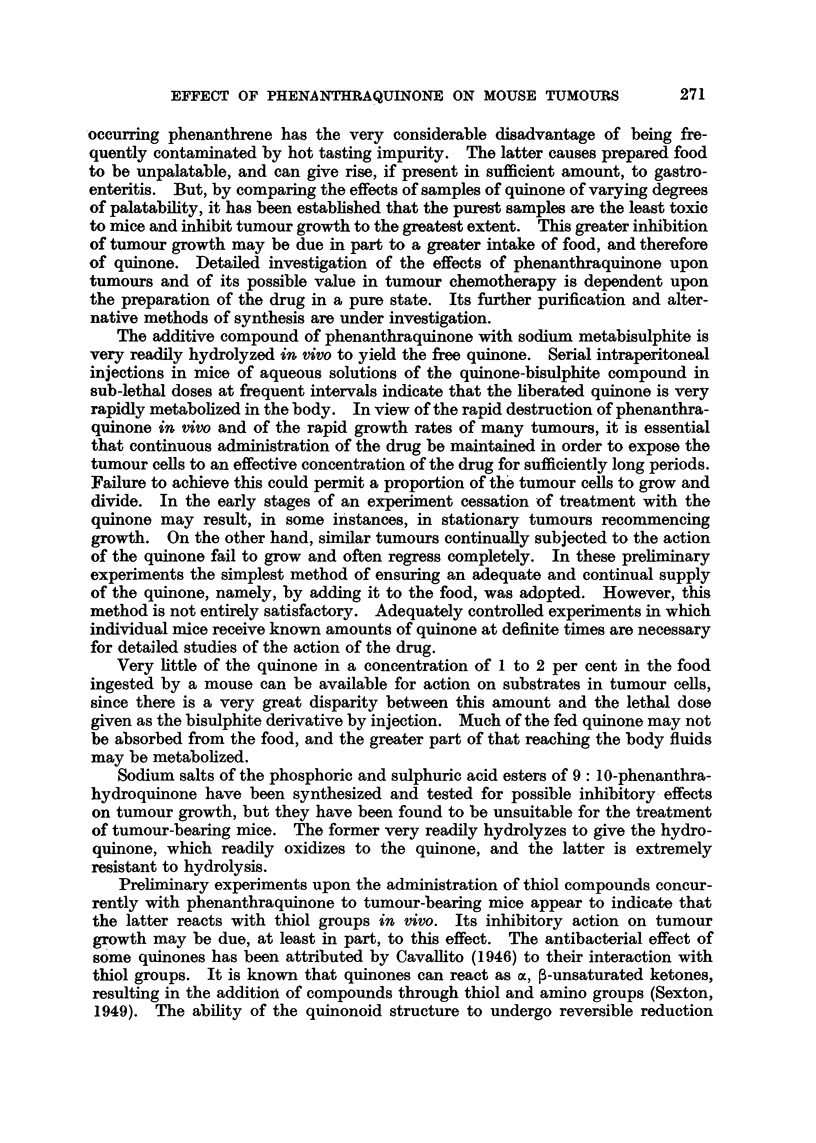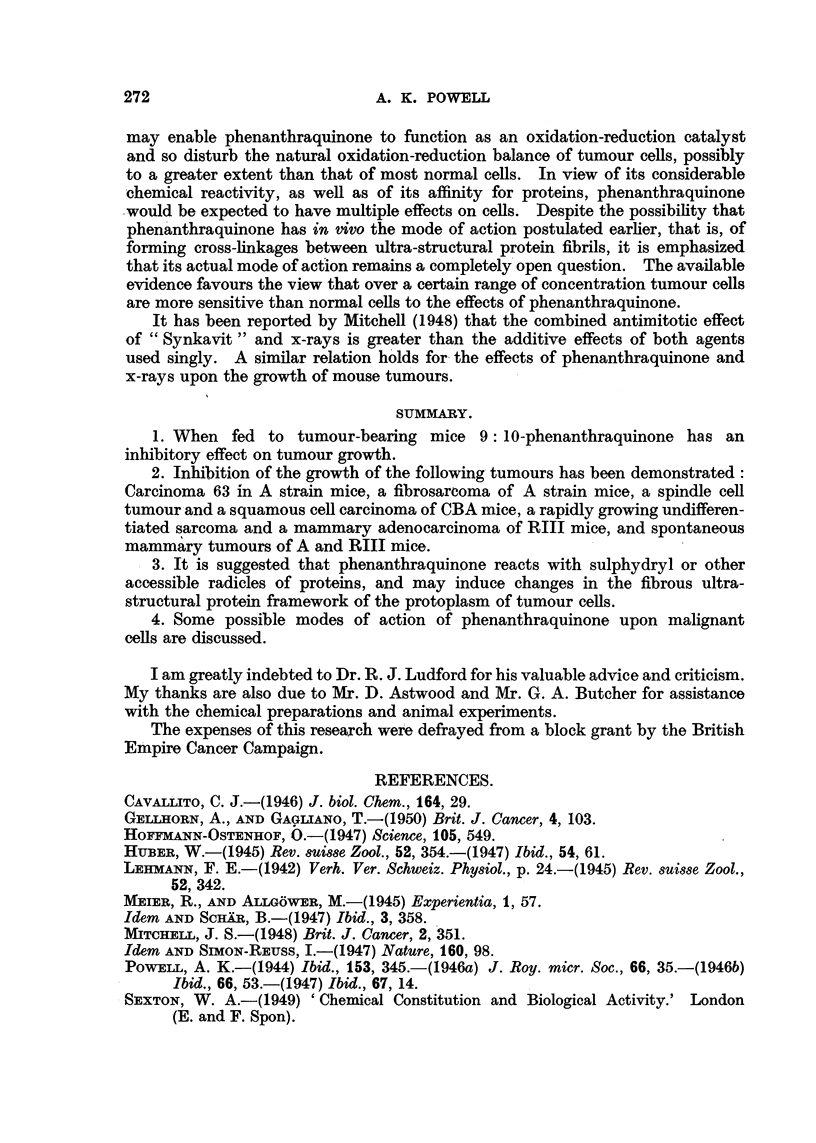# The Inhibitory Effect of 9: 10-Phenanthraquinone upon Tumour Growth in Mice

**DOI:** 10.1038/bjc.1951.27

**Published:** 1951-06

**Authors:** A. K. Powell


### THE INHIBITORY EFFECT OF 9 : 10-PHENANTHRAQUINONE UPON TUMOUR GROWTH IN MICE.

A. K. POWELL.

*From the Department of Experimental Pathology, Mount Vernon Hospital, Northwood, Middlesex.*

Received for publication April 30, 1951.

IN previous communications (Powell, 1944, 1946*a*, 1946*b*, 1947) it has been suggested that many of the typical properties of tumour cells can be explained by the occurrence of a derangement of the submicroscopic protein framework of their protoplasm. It was also suggested that the growth of malignant cells might be inhibited by chemical compounds able to link together the ultra-structural protein fibrils of tumour cells, and thus simulate the arrangement of comparable fibrils in normal cells.

Disulphide bridges constitute one of the more important classes of inter-connections between the fibrils of native proteins. The rupture of disulphide bridges may result in paired thiol groups which can be interlinked by quinones. Quinones can also cross-link protein fibrils by combining with amino and imino groups (Sexton, 1949). In an earlier communication (Powell, 1944) it was reported that certain quinones, and other compounds with a comparable effect on proteins, inhibited the growth of Twort carcinomas in mice.

Quinones have been reported to act as mitotic poisons on fibroblasts grown in tissue culture (Meier and Allgöwer, 1945 ; Meier and Schär, 1947) and on the cells of developing eggs of the fresh-water annelid *Tubifex* (Lehmann, 1942, 1945 ; Huber, 1945, 1947). Mitchell and Simon-Reuss (1947) have described the inhibitory effect of tetrasodium 2-methyl-1 : 4-naphthohydroquinone (" Synkavit ") on normal and malignant cells grown *in vitro*. Mitchell (1948) has also reported increased radiosensitivity in tumours of patients concurrently treated with " Synkavit." However, Gellhorn and Gagliano (1950) subsequently claimed that " Synkavit " had no appreciable inhibitory effect on the growth of transplantable tumours in mice and rabbits. The bacteriostatic effects of quinones are well known (Hoffmann-Ostenhof, 1947).

In the present series of experiments 9 : 10-phenanthraquinone was used as being the simplest readily available quinone likely to show a tumour-inhibiting action without being too toxic for continued administration to experimental animals.

### MATERIALS AND METHODS.

### *Preparation of* 9 : 10-*phenanthraquinone.*

The phenanthraquinone used in the experiments described was prepared by oxidizing phenanthrane in glacial acetic acid solution with chromium trioxide. The reaction product was allowed to cool, diluted with water and the solids collected by filtration. The crude quinone was well washed with water and extracted with sodium metabisulphite solution. The quinone was recovered from the filtered bisulphite solution by precipitation with sodium carbonate or sulphuric acid. Further purification was carried out by repeated formation of the additive bisulphite compound and subsequent precipitation as above. Chloroform or benzene solutions of the purified quinone were then thoroughly extracted with cold dilute sodium carbonate solution, washed with water and dried over anhydrous sodium sulphate. The filtered dry quinone solution was passed through a column of adsorbent alumina and the quinone recovered by distilling off the solvent and washing with ether. It was then recrystallized from acetone.

Prepared by this method, phenanthraquinone contains a variable amount of impurity with a hot burning taste. However, preparations of phenanthraquinone of sufficient purity for preliminary experimental trials on animal tumours have been made by the method described above.

*Administration of phenanthraquinone.*

Preliminary experiments had shown the necessity of continuous administration of quinone to obtain optimum inhibition of tumour growth. Finely ground quinone was therefore incorporated in the food given to treated mice. This food was made up in the proportions : powdered rat cake 80 g., plain flour 20 g., quinone as desired, tap water 50 ml. The dry ingredients, including the quinone, were thoroughly mixed, the water then added and the mixture worked into a stiff dough, which was rolled and cut into small cubes. These were gently dried overnight at a moderate heat. Freshly prepared food was given in excess to treated mice each day.

Control experiments with prepared food lacking the quinone showed that it maintained the health and growth of mice, and that their tumours grew at the same rates as those of mice fed on the ordinary laboratory diet of whole rat cake cubes.

*Experimental animals.*

In all the experiments inbred strains of mice were used. With the exception of Carcinoma 63, which was grown in Strong A strain mice, the tumour strains were homologous with the hosts. The following transplantable tumours were used : Carcinoma 63, a fibrosarcoma (Ac) of Strong A mice, a spindle cell tumour and a squamous cell carcinoma of CBA mice, a rapidly growing undifferentiated sarcoma (Rb) and a mammary adenocarcinoma of RIII mice.

The tumours were grafted subcutaneously in the right flanks of the mice, in each experiment the implants being portions taken from one tumour. In each experiment mice with actively growing tumours were divided into groups, control and experimental. The sizes of the tumours were then recorded, and the mice to be treated placed immediately on the diet containing quinone. The tumours of the mice in each group were measured at intervals, and after the last measurements the tumours from both the treated and control mice were removed for histological study.

### EXPERIMENTAL RESULTS.

The inhibitory effect of food containing 1 to 2 per cent phenanthraquinone upon the growth of Carcinoma 63 is illustrated in the first three charts (Fig. 1, 2,

3). In the first experiment (Fig. 1) the grafts grew more quickly than is usual for this strain of tumour. The sizes of the tumours at the beginning of this experiment were also appreciably larger than in the two other experiments.

In the first experiment (Fig. 1) the majority of the tumours of the mice in both treated groups grew during the early days of treatment, but later showed little growth. Tumour No. 13 did, however, show considerable growth after an initial inhibition.

FIG. 1.—Effect of 1 per cent phenanthraquinone upon the growth of Carcinoma 63 in A strain mice. In all the text-figures the horizontally placed numbers indicate days after inoculation, and the concentration of phenanthraquinone in the food is given as a percentage of the combined weights of flour and rat cake powder.

The quinone used in this experiment possibly contained a trace of the hot tasting impurity, but treated mice showed no harmful effects from it. The growth-inhibitory effect of the drug is evident from a study of the chart. It became more marked with the duration of treatment. In this experiment the control mice were fed upon food prepared from rat cake, flour and water. Ordinary rat cake was given to control animals in all the following experiments.

FIG. 3.

FIG. 2.

FIG. 2 and 3.—Two experiments showing the effect of 2 per cent phenanthraquinone upon the growth of Carcinoma 63 in A strain mice.

The charts shown in Fig. 2 and 3 demonstrate the similarity of the results obtained in different experiments with a particular strain of tumour when the rate of tumour growth is approximately the same, and the concentration of the drug is constant. They should be contrasted with that in Fig. 1, in which the growth of the control tumours is appreciably faster. Here, with a higher concentration of the drug, smaller initial sizes and slower intrinsic growth rates of the tumours, the inhibitory effects of the treatment are greater than in

FIG. 4.—Effect of 2 per cent phenanthraquinone upon the growth of a fibrosarcoma of A strain mice.

the first experiment (Fig. 1). At the end of the second experiment (Fig. 2) one tumour, No. 8, had grown very slightly, three others were stationary, and the remaining three were not visible externally. The results of the other experiment with food containing 2 per cent quinone (Fig. 3) are of the same order.

The remaining charts (Fig. 4, 5, 6) demonstrate the effects of phenanthraquinone upon the growth of tumours homologous with their hosts. At a concentration of 2 per cent in the food, the quinone significantly inhibited the growth of a rapidly growing fibrosarcoma of A strain mice (Fig. 4), a spindle cell tumour of CBA strain mice (Fig. 5), and a very rapidly growing sarcoma of RIII strain mice (Fig. 6). Complete regressions were observed in two of these latter experiments.

Considering the experimental results as a whole it appears that inhibition of tumour growth is greatest when small tumours are treated and the intrinsic growth rate is slow. After transplanted tumours have attained a certain size, varying with the strain, the growth-inhibiting effect of phenanthraquinone is

Fig. 5.—Effect of 2 per cent phenanthraquinone upon the growth of a spindle cell tumour of CBA strain mice.

considerably less. The inhibition of several spontaneous mammary tumours of A and RIII strains of mice by the quinone was comparable with that of the transplanted tumours. Also, the growth of a transplantable mammary adenocarcinoma of RIII mice and a transplantable squamous cell carcinoma of CBA mice was strongly inhibited. Thus the growth of a variety of different tumours can be inhibited by phenanthraquinone under the conditions described.

Samples of phenanthraquinone containing sufficient impurity to make it unpalatable cause loss of weight of the treated animals. Gastro-enteritis sometimes results in such mice, but affected mice may recover completely from the toxic effects. Inhibition of tumour growth precedes signs of toxicity in the host mice. Treated mice in which tumours are no longer palpable have been kept 6 to 8 weeks with no resumption of tumour growth.

FIG. 6.—Effect of 2 per cent phenanthraquinone upon the growth of an undifferentiated sarcoma of RIII strain mice.

### DISCUSSION.

The results of the experiments described demonstrate that the growth of animal tumours can be inhibited by the administration of phenanthraquinone. The quinone prepared by the methods used in the present work from naturally occurring phenanthrene has the very considerable disadvantage of being frequently contaminated by hot tasting impurity. The latter causes prepared food to be unpalatable, and can give rise, if present in sufficient amount, to gastroenteritis. But, by comparing the effects of samples of quinone of varying degrees of palatability, it has been established that the purest samples are the least toxic to mice and inhibit tumour growth to the greatest extent. This greater inhibition of tumour growth may be due in part to a greater intake of food, and therefore of quinone. Detailed investigation of the effects of phenanthraquinone upon tumours and of its possible value in tumour chemotherapy is dependent upon the preparation of the drug in a pure state. Its further purification and alternative methods of synthesis are under investigation.

The additive compound of phenanthraquinone with sodium metabisulphite is very readily hydrolyzed *in vivo* to yield the free quinone. Serial intraperitoneal injections in mice of aqueous solutions of the quinone-bisulphite compound in sub-lethal doses at frequent intervals indicate that the liberated quinone is very rapidly metabolized in the body. In view of the rapid destruction of phenanthraquinone *in vivo* and of the rapid growth rates of many tumours, it is essential that continuous administration of the drug be maintained in order to expose the tumour cells to an effective concentration of the drug for sufficiently long periods. Failure to achieve this could permit a proportion of the tumour cells to grow and divide. In the early stages of an experiment cessation of treatment with the quinone may result, in some instances, in stationary tumours recommencing growth. On the other hand, similar tumours continually subjected to the action of the quinone fail to grow and often regress completely. In these preliminary experiments the simplest method of ensuring an adequate and continual supply of the quinone, namely, by adding it to the food, was adopted. However, this method is not entirely satisfactory. Adequately controlled experiments in which individual mice receive known amounts of quinone at definite times are necessary for detailed studies of the action of the drug.

Very little of the quinone in a concentration of 1 to 2 per cent in the food ingested by a mouse can be available for action on substrates in tumour cells, since there is a very great disparity between this amount and the lethal dose given as the bisulphite derivative by injection. Much of the fed quinone may not be absorbed from the food, and the greater part of that reaching the body fluids may be metabolized.

Sodium salts of the phosphoric and sulphuric acid esters of 9 : 10-phenanthrahydroquinone have been synthesized and tested for possible inhibitory effects on tumour growth, but they have been found to be unsuitable for the treatment of tumour-bearing mice. The former very readily hydrolyzes to give the hydroquinone, which readily oxidizes to the quinone, and the latter is extremely resistant to hydrolysis.

Preliminary experiments upon the administration of thiol compounds concurrently with phenanthraquinone to tumour-bearing mice appear to indicate that the latter reacts with thiol groups *in vivo*. Its inhibitory action on tumour growth may be due, at least in part, to this effect. The antibacterial effect of some quinones has been attributed by Cavallito (1946) to their interaction with thiol groups. It is known that quinones can react as $\alpha$, $\beta$-unsaturated ketones, resulting in the addition of compounds through thiol and amino groups (Sexton, 1949). The ability of the quinonoid structure to undergo reversible reduction may enable phenanthraquinone to function as an oxidation-reduction catalyst and so disturb the natural oxidation-reduction balance of tumour cells, possibly to a greater extent than that of most normal cells. In view of its considerable chemical reactivity, as well as of its affinity for proteins, phenanthraquinone would be expected to have multiple effects on cells. Despite the possibility that phenanthraquinone has *in vivo* the mode of action postulated earlier, that is, of forming cross-linkages between ultra-structural protein fibrils, it is emphasized that its actual mode of action remains a completely open question. The available evidence favours the view that over a certain range of concentration tumour cells are more sensitive than normal cells to the effects of phenanthraquinone.

It has been reported by Mitchell (1948) that the combined antimitotic effect of "Synkavit" and x-rays is greater than the additive effects of both agents used singly. A similar relation holds for the effects of phenanthraquinone and x-rays upon the growth of mouse tumours.

### SUMMARY.

1. When fed to tumour-bearing mice 9 : 10-phenanthraquinone has an inhibitory effect on tumour growth.

2. Inhibition of the growth of the following tumours has been demonstrated : Carcinoma 63 in A strain mice, a fibrosarcoma of A strain mice, a spindle cell tumour and a squamous cell carcinoma of CBA mice, a rapidly growing undifferentiated sarcoma and a mammary adenocarcinoma of RIII mice, and spontaneous mammary tumours of A and RIII mice.

3. It is suggested that phenanthraquinone reacts with sulphydryl or other accessible radicles of proteins, and may induce changes in the fibrous ultrastructural protein framework of the protoplasm of tumour cells.

4. Some possible modes of action of phenanthraquinone upon malignant cells are discussed.

I am greatly indebted to Dr. R. J. Ludford for his valuable advice and criticism. My thanks are also due to Mr. D. Astwood and Mr. G. A. Butcher for assistance with the chemical preparations and animal experiments.

The expenses of this research were defrayed from a block grant by the British Empire Cancer Campaign.

### REFERENCES.

CAVALLITO, C. J.—(1946) *J. biol. Chem.*, **164**, 29.

GELLHORN, A., AND GAGLIANO, T.—(1950) *Brit. J. Cancer*, **4**, 103.

HOFFMANN-OSTENHOF, O.—(1947) *Science*, **105**, 549.

HUBER, W.—(1945) *Rev. suisse Zool.*, **52**, 354.—(1947) *Ibid.*, **54**, 61.

LEHMANN, F. E.—(1942) *Verh. Ver. Schweiz. Physiol.*, p. 24.—(1945) *Rev. suisse Zool.*, **52**, 342.

MEIER, R., AND ALLGÖWER, M.—(1945) *Experientia*, **1**, 57.

*Idem* AND SCHÄR, B.—(1947) *Ibid.*, **3**, 358.

MITCHELL, J. S.—(1948) *Brit. J. Cancer*, **2**, 351.

*Idem* AND SIMON-REUSS, I.—(1947) *Nature*, **160**, 98.

POWELL, A. K.—(1944) *Ibid.*, **153**, 345.—(1946*a*) *J. Roy. micr. Soc.*, **66**, 35.—(1946*b*) *Ibid.*, **66**, 53.—(1947) *Ibid.*, **67**, 14.

SEXTON, W. A.—(1949) 'Chemical Constitution and Biological Activity.' London (E. and F. Spon).